# High BMI and Low Muscular Fitness Predict Low Motor Competence in School-Aged Children Living in Low-Resourced Areas

**DOI:** 10.3390/ijerph18157878

**Published:** 2021-07-25

**Authors:** Evi Verbecque, Dané Coetzee, Gillian Ferguson, Bouwien Smits-Engelsman

**Affiliations:** 1 Rehabilitation Research Centre (REVAL), Hasselt University, Agoralaan Building A, 3590 Diepenbeek, Belgium; 2 Physical Activity, Sport and Recreation, Faculty Health Sciences, North-West University, Potchefstroom 2520, South Africa; Dane.Coetzee@nwu.ac.za (D.C.); bouwienengelsman@icloud.com (B.S.-E.); 3 Department of Health & Rehabilitation Sciences, Faculty of Health Sciences, Cape Town University, Cape Town 7701, South Africa; gillian.ferguson@uct.ac.za

**Keywords:** motor skills, motor competence, muscular fitness, age, socio-economic status, overweight, obesity

## Abstract

Childhood obesity is a relatively new problem for Sub-Saharan developing countries. Especially in children with a low socioeconomic background, the link between motor competence, muscular fitness, and body mass index (BMI) remains poorly investigated. Due to the interrelatedness of BMI and physical fitness, the aim of this study is to determine the predictive value of these factors in relation to low motor competence in school-aged children living in low-resourced areas. Motor competence and physical fitness were assessed in 1037 school-aged Ghanaian and South African children using the Performance and Fitness test battery (PERF-FIT). “Low motor competence” was predicted using odds ratios calculated from backward logistic regression analyses. Low motor competence was less prevalent in Ghanaian children (3.7–11.1%) compared to the South African children (21.9–24.2%). Increased BMI and decreased muscular fitness predicted low motor competence in both Ghanaian and South African children. For example, the chance for a Ghanaian child to have low static balance increased by 22.8% (OR = 1.228, *p* < 0.001) with a 1-point increase in BMI, whereas this decreased by 30.0% (OR = 0.970, *p* < 0.001) with a 10-cm increase on the standing long jump. In the case of the South African children, if their BMI increased by 1 point, the chance for those children of having low static balance increased by 7.9%, and if their SLJ performance decreased by 10 cm, their chance of low performance increased by 13%. Clearly, motor competence is associated with both BMI and muscular fitness. Policy makers can use this information to counteract the establishment of childhood obesity by promoting weight control through physical activity and stimulating motor competence at school.

## 1. Introduction

Overweight and obesity were previously perceived as typical problems of developed countries, but currently in low- and middle-income countries, a relatively rapid transition from underweight to overweight and obesity is occurring [1]. The increasing prevalence of overweight and obesity in western and southern Africa is remarkable [1]. For example, between 2000 and 2013, overweight and obesity increased from 11 to 19% in southern African countries, whereas the prevalence of stunted children remained almost the same [2]. In 2020, approximately 17% of the children between age 5 and 19 in South Africa presented with overweight and obesity [3], which was estimated at 19% for all children under age 19 in Ghana [4]. In general, it is often reported that more girls tend to have overweight or obesity compared to boys [5].

To reduce obesity, good nutrition and physical health are vital [6]. Recent systematic reviews and meta-analyses showed that physical health is associated with physical activity and physical fitness [7,8] and that physical activity on its own is associated with gross motor competence, a child’s ability to execute different gross motor acts necessary for participating in everyday activities and sports [7,9]. Children with low levels of gross motor competence tend to be less active and have lower levels of cardiorespiratory [7,10] and muscular fitness [11,12]. Indeed, the model by Stodden et al. (2008), shows the interrelationship between (perceived) motor competence, health related fitness, and physical activity and how it influences the risk of developing obesity, thereby causing either a positive or a negative spiral of (dis)engagement [13]. Since then, several studies have investigated whether and how overweight and obesity are related to low motor competence in different age groups [14,15,16] and by tracking child development longitudinally [17,18,19,20]. Previous studies showed that, especially in overweight and obese children, low motor competence is reported frequently [14,15,17,18,19,20]. The evolution of gross motor competence in children is related to the child’s BMI status [14,15,17,18,19]. For instance, a high BMI in children as young as 5 years old contributes to declines in gross motor competence in the years thereafter [20]. The reciprocal association, where children with lower motor competence have a higher risk of being overweight, is not unambiguous [18,20]. For instance, Lima et al. (2019) [18] did find poor motor competence at age 6 to be associated with fatness during childhood, whereas Cheng et al. (2016) [20] did not find poor motor competence in 5-year-olds to be related to obesity or increases in BMI at age 10. Furthermore, not only do children with overweight and obesity have significant lower levels of gross motor competence, physical activity, and physical fitness, they also exhibit difficulties in executive functioning, e.g., planning and control of movement [21]. Furthermore, structural differences in cerebellar peduncles have been identified in obese children, indicating obesity is accompanied by alterations in white matter organization [22].

Previous research has also shown that gross motor competence is associated with socioeconomic status (SES) [23,24,25]. Children living in disadvantaged circumstances have fewer chances of participating in organized physical activity and sports [9], which hampers the development of such specific skills. Furthermore, children living in low- and middle-income countries also frequently suffer from malnutrition [26]. The term malnutrition is usually applied in cases of underweight or undernutrition, but can also refer to the other end of the BMI spectrum. Because overweight and obesity are a relatively new problem for developing countries, few studies have been performed to investigate the link between motor competence, muscular fitness, and BMI in children with a low SES background [17,18,27,28,29]. Currently available reports mainly focus on comparing motor competence and physical fitness among different BMI groups, showing overweight and obese children do have lower motor competence compared to normal weight peers [27,28,29]. However, due to the interrelatedness of BMI and physical fitness, determining the predictive value of these factors with respect to low motor competence could improve insights into how it can be managed, which is relevant information for policy makers.

Recently, a new test for physical fitness and gross motor competence was specifically developed for use in low-resourced areas [30]. The Performance and Fitness test battery (PERF-FIT) evaluates movement skills, musculoskeletal fitness, and agility and power, and its feasibility and content validity [30], reliability [31], and construct validity [32] have been proven in school-aged children. Because SES is known to be an influencing factor for motor competence, the first aim of this study is to investigate the prevalence of low motor competence in children living in low-resourced areas in Ghana and South Africa. Furthermore, locomotor and stability skills are particularly associated with body composition and muscular fitness [7]. Therefore, the second aim of this study is to determine the contribution of body composition and muscular fitness to the prediction of low motor competence in these children.

## 2. Materials and Methods

### 2.1. Participants

Children aged 6 to 12 years were recruited from four primary schools in South Africa, (two in the Western Cape and two in North West Province) and from five primary schools in Ghana (four near Accra and one in the Eastern region of Ghana). Twelve hundred children were invited to participate in the study and were recruited through stratified sampling, spread over nine schools (located in different SES areas: four schools low SES; three schools low-middle SES; one school middle SES; and one school located in high-middle SES). The participating schools were situated in low SES areas and were recruited through an indirect network of the researchers. Once the schools gave consent to participate, the children were randomly selected. The children participated in this cross-sectional study after their parents provided written informed consent. Data collection took place between January and September 2019. The study protocol was approved by the local ethical committees (NWU-00491-19-A1, HREC Ref 598/2019, and GHS-ERC 084/04/19).

The parent(s) filled in the child physical activity readiness questionnaire [27]. Children were excluded from the sample if they had: (i) a formal diagnosis that would significantly impede motor performance as reported by the parents, (ii) refused testing, or (iii) incomplete test results due to absence from school during test administration. Neither children nor legal guardians received financial compensation for their participation.

### 2.2. Measurements

Weight (BF 511 Omron, measured to nearest 0.1 kg) and height (a wall-mounted tape measure, measured to the nearest 0.1 cm) were recorded to calculate BMI (kg/m^2^). Next, the PERF-FIT, a valid and reliable test to measure movement skills, musculoskeletal fitness, and agility in children this age in low resourced communities [24,25,26,28], was administered. The test consists of two subscales. The power and agility subscale comprises three agility items (running, stepping, and side jumping) and two explosive power items (overhand throw and Standing Long Jump (SLJ)). Although the SLJ is considered a fundamental movement skill, due to its strong relationship with other measures for lower extremity power [33,34], it represents a general index of muscular fitness in youth [29,30]. The motor skills performance subscale contains five series of tasks (referred to as skill item series (SIS)) with increasing difficulty: (1) throw and catch, (2) bounce and catch, (3) static balance and dynamic balance, (4) jumping, and (5) hopping. For studying the relation with body composition, muscular fitness, and motor competence, the SLJ, static balance, dynamic balance, jumping, and hopping items are reported in this study and are therefore described in more detail in Table 1.

### 2.3. Statistical Analysis

Statistical analyses were performed with SPSS 27.0 for windows.

The sample was described using demographic data (age, sex), anthropometric data (weight, height, BMI), and muscular fitness data from the PERF-FIT. The Shapiro-Wilk test was used to check for normality. The prevalence of overweight and obesity was determined using the BMI-for-age-and-sex percentiles defined by Cole et al. (2012) [36] and expressed as a percentage of the entire sample. To compare the children’s age, BMI, and muscular fitness per country (Ghana versus South Africa) in the entire sample and within the age groups, a one-way analysis of variance (ANOVA) was used and a two-tailed Chi-squared test for the distribution of the sexes.

Age and sex may or may not have an influence on motor competence [7], depending on the applied outcome. These factors were therefore considered in the analyses. To explore age-related differences in the motor competence outcome variables, the Kruskal-Wallis test was applied.

The distribution of the dependent variables was skewed, such that the use of cut-off values, to identify low performance, were clinically more relevant as opposed to using median values or interquartile ranges (IQR). Therefore, to predict low motor competence, the dependent variables were dichotomized (0 = normal performance, 1 = low performance) using percentile 15 (P15) as a cut-off value. This cut-off value was chosen as motor scales usually apply the 15th percentile as a cut-off value to decide whether a motor performance is below average for age because this represents performances 1SD away from the mean. Because of the age effect on all PERF-FIT SIS (static balance SIS: H(5) = 100.933, *p* < 0.001; dynamic balance SIS: H(5) = 281.148, *p* < 0.001; Jumping and hopping SIS: H(5) = 245.370, *p* < 0.001), age-specific percentile values were used for dichotomization, hereafter referred to as the motor competence groups (normal versus low motor competence).

Subsequently, a two-tailed Chi-square test revealed that the distribution of Ghanaian and South African children across the motor competence groups was significantly different for all dependent variables (static balance SIS: X^2^ = 89.893, *p* < 0.001; dynamic balance: X^2^ = 28.637, *p* < 0.001; jumping and hopping SIS: X^2^ = 36.459, *p* < 0.001). Therefore, the next analyses were performed separately per country (Ghana versus South Africa). As such, a two-tailed Chi-square test was used to establish whether the sex and SES distribution and the percentage of children living in urban or rural areas were similar between each motor competence group. The Mann-Whitney-U test was used to compare the BMI and SLJ performance in the normal (>P15) versus the low (≤P15) motor competence groups. Next, backward conditional logistic regression analysis was applied to calculate odds ratios. The predicted value was “low motor competence” for the static balance, dynamic balance, and jumping and hopping, with BMI (continuous) and SLJ performance (continuous) as predictors. Sex was not added to the model because the Chi-square test was not significant for the static and dynamic balance SIS. There were differences between boys and girls regarding jumping and hopping performance (see Figure S1 and Table S1), and their distribution was also significantly different across the motor competence groups. Furthermore, for the jumping and hopping SIS, the residential area was also significantly differently distributed across the groups. Hence, sex and residential area were added to the model in addition to BMI and SLJ. However, sex was not a significant predictor in the model. Odds ratios (ORs) were derived from the exponential β value and the corresponding 95% CI. Significance was set at *p* < 0.05.

## 3. Results

### 3.1. Participants

**Figure 1:**
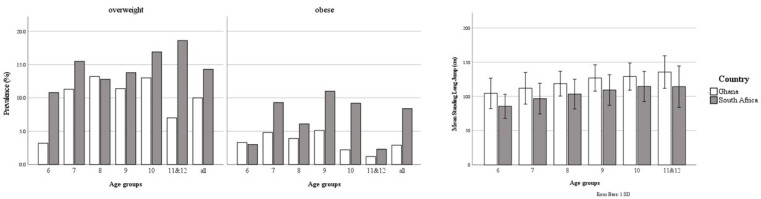
The prevalence of overweight and obesity (left) and the mean standing long jump performance (right) classified according to country.

Approximately eighty-seven percent (1040/1200) of the invited children participated in this study. Three 5-year-olds were excluded because they were too young, leaving 1037 children for data analysis. Table 2 contains a detailed description of the entire sample and the different age groups. An equal number of boys and girls participated in both countries (X^2^ = 0.046, *p* = 0.830). The Ghanaian children were significantly older (F_1,1035_ = 121.146, *p* < 0.001), had a lower BMI (F_1,1035_ = 23.149, *p* < 0.001), and higher muscular fitness (F_1,1033_ = 227.268, *p* < 0.001) (Table 2). The combined prevalence over the age groups of overweight and obesity was 12.9% in the Ghanaian sample, of which 47% lived in urban area, and the prevalence of overweight and obesity was 22.6% in the South African sample, of which 71% lived in urban area. The prevalence of underweight was 21.5% and 7.8%, for Ghana and South Africa, respectively (Figure 1).

### 3.2. PERF-FIT Performance

The prevalence of low motor competence was significantly lower in the Ghanaian sample compared to the South African children for all motor competencies. Table 3 shows the distribution of the children in motor competence groups and the medians and IQR for age, BMI, and SLJ performance in each motor competence group. For each skill, fewer Ghanaian children showed low motor competence compared to the South African children and, in both countries, the motor competence groups consisted of a similar number of boys and girls (Ghana: X^2^ = 0.504, *p* = 0.495; South Africa: X^2^ = 0.378, *p* = 0.543). For both the Ghanaian and South African children, BMI and SLJ performance were significant predictors for low motor competence in static balance, dynamic balance, and jumping and hopping.

#### 3.2.1. Static Balance SIS

**Figure 2:**
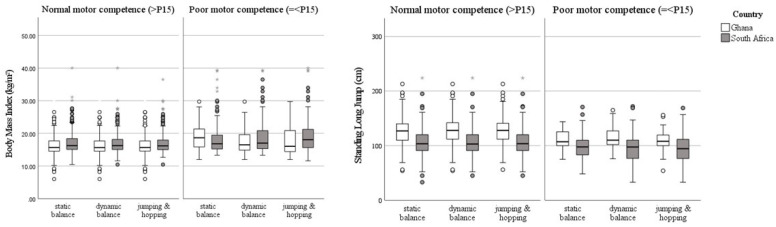
Boxplots depicting the distribution of body mass index and standing long jump performance per motor competence group. * means outliers.

In both the Ghanaian and South African sample, the group with a low static balance performance had a significantly higher median BMI (Ghanaian group: U = 6606, z = 3.059, *p* = 0.002; South African group: U = 28,519, z = 2.228, *p* = 0.026) and lower median SLJ performance (Ghanaian group: U = 2916, z = −2.784, *p* = 0.005; South African group: U = 20,181.5, z = −3.232, *p* = 0.001) compared to those with a normal static balance (Table 3; Figure 2). Table 4 shows the probability of low static balance based on changed BMI and SLJ for both groups in terms of odds ratios.

#### 3.2.2. Dynamic Balance SIS

In both the Ghanaian and South African sample, the group with a low dynamic balance performance had a significantly higher median BMI (Ghanaian group: U = 15,173, z = 2.126, *p* = 0.033; South African group: U = 30,029, z = 3.397, *p* < 0.001) and lower median SLJ performance (Ghanaian group: U = 8556.5, z = −4.171, *p* < 0.001; South African group: U = 19,364.5, z = −3.656, *p* < 0.001) compared to those with a normal dynamic balance (Table 3; Figure 2). Table 4 shows the probability of low dynamic balance based on changed BMI and SLJ for both groups in terms of odds ratios.

#### 3.2.3. Jumping and Hopping SIS

In the Ghanaian sample, the group with a low jumping and hopping competence had a lower median SLJ performance (U = 5403.5, z = −5.028, *p* < 0.001) but similar BMI compared to normal motor competence group (Table 3; Figure 2). The South African children with a low jumping and hopping performance had both a higher median BMI (U = 30,855, z = 5.064, *p* < 0.001) and a lower median SLJ performance (U = 18,019.5, z = −3.801, *p* < 0.001) compared to those with a normal jumping and hopping performance (Table 3; Figure 2). Table 4 shows the probability of low jumping and hopping performance based on living in an urban area or changed BMI and SLJ for both groups in terms of odds ratios.

## 4. Discussion

The aim of this study was to investigate the prevalence of low motor competence in Ghanaian and South African school-aged children living in low-resourced areas and subsequently to establish the extent to which body composition and muscular fitness predict low gross motor competence. Our study showed that: (1) Significantly fewer Ghanaian children had a low motor competence compared to South African children; (2) In both samples, low motor competence in static and dynamic balance as well as jumping and hopping can be predicted by both body composition (BMI, ranging between 7.9–22.8% per increasing BMI unit) and muscular fitness (SLJ, ranging between 13–35% per decreasing 10 cm); and (3) Children living in urban areas are more likely to have low jumping and hopping performance compared to those living in rural areas.

In line with the literature, BMI was related to all motor skills tested. In general, except for the 6-year-olds where the mean is below P50, the children in the current sample have a slightly higher BMI than those in the growth curves of the World Health Organization [32]. First, Ghanaian children have a lower BMI compared to the South African children, and there is a smaller portion with overweight and obesity (Ghana: 12.9%; South Africa: 22.6%). Additionally, overweight and obesity tended to be more prevalent in the older age groups, especially in the South African sample. Nevertheless, for both samples the chance of belonging to the low motor competence group increased significantly if their BMI increased by 1 point (ranging from 7.9% to 22.8%). These findings confirm the inverse relationship between BMI and motor skill competence [7]. Previous research has shown that older children with high BMI levels exhibit lower motor competence compared to younger children, stressing the enlarging impact of growing older on the reciprocal association between motor competence and overweight [14,15,19]. Interestingly, the Ghanaian children were older than the South African children and also showed less overweight and obesity. Hence, increasing BMI has a much larger impact on motor competence, as shown by higher odds ratios in this specific group. This may suggest that the negative spiral between motor competence, BMI, and muscular fitness has not reached a similar level in both countries.

Furthermore, we found a link between muscular fitness and motor competence. Although the impact of the individual factors is important, both BMI and SLJ performance additively predicted low performance on all assessed motor skills. This is not surprising as physical fitness or strength and weight are associated with each other [33]. A heavier body is more difficult to move and control during functional movements as it requires relatively more power [7], a feature limited in these children, especially the South African sample. Similar to a global trend of children becoming less active and overweight, physical fitness in our African samples is lower compared to previous studies [37]. Especially, the Ghanaian sample showed that when children have lower muscular fitness, the impact on their motor competence is detrimental. Similar to what the Stodden model [13] suggests, our results indicate that higher levels of muscular fitness may protect a child from presenting with low motor competence, which is why attention should be given to this factor as well [33,37]. Indeed, strength training (combined with anaerobic and aerobic exercises) positively influences BMI and adiposity in children with overweigh and obesity [38,39].

Investigating these specific groups of children provided a unique opportunity to map the impact of children living in different environmental circumstances. Interestingly, in both countries, living in urban areas predicts low motor competence for jumping and hopping. This is not surprising as such skills require room for movement. South African children living in informal settlements in urban areas do not have many opportunities to participate in organized sports activities and play outside because of the increased crime and unsafe environment [40,41]. Subsequently, for safety purposes, approximately 40% of the South African children do not walk to school, but go by taxi busses, decreasing their daily physical activities [40]. Although many Ghanaian children also live in low resourced areas, they do not face such unsafe environments, allowing them to play outside, and most children in our sample walk to school. Hence, it is not surprising that a significantly larger number of South African children have low motor skill related fitness compared to their Ghanaian peers. The current results indicate that these physical activity restrictions also have large implications for the children’s body composition and muscular fitness. Nevertheless, even for the Ghanaian children, living in urban areas may have a negative impact on motor competence, indicating that both our samples would benefit from more opportunities that allow practicing movement skills. However, this would require safe environments, such as public parks, playgrounds, community centers, and walking trails/hiking areas, where the children can be active and keep practicing.

Studies performed in developed countries, which have been dealing with the impact of overweight and obesity on child development for several decades, have already shown that high BMI at a young age contributes to declines in motor competence at a later age [17,18], but also the reverse impact has been shown [18]. Therefore, early childhood interventions targeting weight control can positively impact motor competence [17]. To ensure long-term effects, weight control needs to be targeted, not only through diet, but also by increasing physical activity and physical fitness. Because motor competence in our sample was related to muscular fitness and previous research showed that a certain level of motor competence is required to enjoy and maintain long-term physical activity [12], promoting physical activity and fitness is much needed. Especially children with low balance, such as in our sample, benefit from weight loss and training [21]. These results have important implications for policy makers in both countries targeting overweight. For South African children, the vicious circle of physical inactivity, overweight and obesity, and low motor competence needs to be broken, e.g., more physical activity at school. Although physical education forms part of the life skills curriculum in South Africa, its actual delivery in the schools may be insufficient [40]. For Ghanaian children, the current results show that discontinuation of the gradually increasing prevalence of overweight and obesity in children is imperative and that prevention is in order, which also requires implementation of regular physical education at schools and organized sports activities, next to attention regarding nutrition. Interventions focusing on improving motor skills in children with overweight and obesity have proven to be successful on the body composition, physical fitness, and gross motor competence [42].

### Study Strengths and Limitations

Although several systematic reviews and meta-analyses have been performed on factors that influence motor competence, literature on this topic in children living in low and middle-income regions is scarce. The included sample is large and was recruited in different regions in two African countries, which improves generalizability of the results. A limitation of this study is that the sample was not entirely randomly selected. However, the specific group of children targeted did not allow such a sampling method, and the main aim was to recruit a representative sample. More background on sedentary behavior in our sample would have helped to interpret the data better. Physical fitness and motor competence were both measured using fundamental motor skills, for which some authors [43] argue that a general motor ability could explain the interrelatedness. Nevertheless, motor abilities are task-dependent as the tasks dictate which aspects of motor competence and their underlying body functions, such as muscle strength, coordination, or balance, are being tapped into. Thus, although the standing long jump, used for assessing muscular fitness also requires coordination and balance, this test has been proven to correlate strongly to other measures for lower extremity power [33].

## 5. Conclusions

The present study showed that low motor competence measured with the PERF-FIT can be predicted by increased BMI and decreased muscular fitness. These findings provide policy makers with important knowledge on the current state of overweight and physical fitness in children living in low-resourced Sub-Saharan areas, which can be used to counteract the establishment of childhood obesity by promoting physical activity, preferably at school. Evaluation using the PERF-FIT can therefore be used to plan additional support to stimulate motor competence through physical education and organized sports activities at school in children living in low and middle-income countries.

## Figures and Tables

**Table 1:** Description of PERF-FIT standing long jump and motor skill item series.

Skill Item Series	Items	Description
**Standing Long jump**	**1**	The child is asked to stand with his/her toes behind a starting line and to jump as far as possible without falling [35]. One practice trial with submaximal force is given, followed by two test trials with 15 s rest between trials. The distance (cm) between the starting line and the heel of the foot that landed closest to the starting line is measured. The best performance is the final result.
**Static balance SIS**	**4**	Four items: (1) standing and hugging the knee for maximum 15 s (left and right), (2) standing and grasping the foot for maximum 15 s (left and right) [35]. Timing started when the knee was hugged or the foot was grasped and stopped if the raised leg touched the standing leg, corrective hops were made, or the child lost balance or fell. Per item, a second trial was allowed if the child performed submaximal during the first trial. The best trial was the final result. The scores of each item were summed, resulting in scores between 0–60 s.
**Dynamic balance SIS**	**6**	(1) walking while hugging a knee, (2) walking while grasping a foot, (3) standing on one leg while moving cans from close to far (left and right), and (4) standing on one leg while moving cans from far to close (left and right) [35]. First, the child was asked to walk slowly in an agility ladder (max 8 steps) while hugging a knee or grasping a foot without touching the borders, stepping outside the borders, or losing balance. For each correctly placed step, a point was awarded. For both items, a second trial was allowed if the child performed submaximal during the first trial. The best trial was the final result. With these two items, a maximum of 16 points could be earned [35]. Afterwards, the child stood on one leg while picking up 4 cans consecutively and moving them from close to far (or the other way around) without moving the stance foot, losing balance, or placing the raised leg on the ground. One point was awarded for each correctly placed can (max 4 points). The children performed this for both legs and in both directions (i.e., 4 items) and could earn a maximum of 16 points. The scores of the dynamic balance items were summed, with scores between 0 and 32 points.
**Jumping and hopping SIS**	**12**	Four jumping items and four hopping items (left and right) [35]. The jumping and hopping SIS consists of four levels of difficulty: jump/hop in each square, in every other square, in every other square over a five-centimeter foam pad, and in every other square over a ten-centimeter foam pad. One point was awarded for each correct jump/hop. For the jumping items a total of 20 points could be earned and for the hopping items 40 points, resulting in a total score varying between 0 and 60 points.

**Table 2:** Description of the sample.

	All Children	Age Groups					
		6	7	8	9	10	11–12
Boys (n)/girls (n)	521/516	44/52	77/82	117/106	103/85	77/80	103/111
Ghanaian (n)/South African (n)	511/526	31/65	62/97	76/147	79/109	92/65	171/43
Age (years, mean (SD))	9.2 (1.7)	6.4 (0.4)	7.4 (0.3)	8.3 (0.3)	9.4 (0.3)	10.4 (0.3)	11.7 (0.5)
Weight (kg, mean (SD))	31.2 (9.9)	22.1 (4.3)	25.9 (6.0)	27.8 (6.8)	31.9 (9.2)	36.2 (10.0)	38.6 (10.1)
Height (cm, mean (SD))	134.5 (11.8)	118.5 (5.7)	125.0 (6.3)	130.0 (7.3)	135.4 (7.4)	142.1 (7.2)	147.1 (9.1)
BMI (kg/m^2^, mean (SD))	16.95 (3.46)	15.64 (2.14)	16.52 (2.97)	16.31 (2.94)	17.25 (3.91)	17.75 (4.00)	17.69 (3.63)
▪ Ghanaian subgroup	16.43 (3.03) ^¥^	15.06 (1.84)	15.64 (2.32) ^◆^	15.75 (3.20) *	16.32 (3.21) ^◆^	16.74 (3.26) ^¥^	17.16 (2.95) ^¥^
○ Overweight (%)	10.0	3.2	11.3	13.2	11.4	13.0	7.0
○ Obese (%)	2.9	3.2	4.8	3.9	5.1	2.2	1.2
▪ South African subgroup	17.46 (3.78) ^¥^	15.92 (2.23)	17.08 (3.21) ^◆^	16.60 (2.77) *	17.92 (4.23) ^◆^	19.17 (4.52) ^¥^	19.80 (5.11) ^¥^
○ Overweight (%)	14.3	10.8	15.5	12.8	13.8	16.9	18.6
○ Obese (%)	8.4	3.0	9.3	6.1	11.0	9.2	2.3
Standing Long Jump (cm, mean (SD))	114 (26)	92 (21)	103 (24)	109 (22)	117 (22.9)	123 (22)	131 (27)
▪ Ghanaian subgroup	126 (24) ^¥^	104 (22) ^¥^	112 (23) ^¥^	119 (18) ^¥^	127 (19) ^¥^	129 (20) ^¥^	136 (24) ^¥^
▪ South African subgroup	103 (24) ^¥^	86 (18) ^¥^	97 (22) ^¥^	103 (22) ^¥^	109 (22) ^¥^	115 (22) ^¥^	114 (31) ^¥^

Legend: BMI: body mass index; underlined values indicate significant differences between the Ghanaian and South African children, significance level: * *p* < 0.05; ^◆^ *p* < 0.01; ^¥^ *p* < 0.001. Underlines values indicate significant differences between the Ghanaian and Sout African children

**Table 3:** Characteristics of the motor competence groups.

	**Static Balance SIS**			
	**Ghana**		**South Africa**	
	**>P15**	**≤P15**	**>P15**	**≤P15**
N (%)	492 (96.3)	19 (3.7)	397 (75.8)	127 (24.2)
Age (years)	10.0 (2.8)	10.1 (3.1)	8.4 (2.0)	8.3 (2.6)
BMI (kg/m^2^)	15.67 (3.17) *	18.64 (6.63) *	16.25 (3.32) *	16.82 (4.28) *
SLJ (cm)	127 (30) *	107 (26) *	103 (29) *	98 (27) *
	**Dynamic Balance SIS**			
	**Ghana**		**South Africa**	
	**>P15**	**≤P15**	**>P15**	**≤P15**
N (%)	454 (88.9)	57 (11.1)	400 (76.2)	125 (23.8)
Age (years)	10.0 (2.8)	10.0 (2.8)	8.3 (2.0)	8.8 (2.6)
BMI (kg/m^2^)	15.65 (3.16) *	16.48 (4.76) *	16.25 (3.04) *	17.03 (5.50) *
SLJ (cm)	128 (30) *	110 (25) *	103 (29)	98 (33)
	**Jumping and hopping SIS**			
	**Ghana**		**South Africa**	
	**>P15**	**≤P15**	**>P15**	**≤P15**
N (%)	468 (91.6)	43 (8.4)	410 (78.1)	115 (21.9)
Age (years)	10.0 (2.8)	10.7 (2.8)	8.4 (2.0)	8.5 (2.7)
BMI (kg/m^2^)	15.67 (3.13)	16.04 (6.57)	16.16 (2.93) *	18.04 (5.6) *
SLJ (cm)	128 (29) *	108 (20) *	104 (29) *	94 (36) *

Legend: Age, BMI, and SLJ are presented as medians and interquartile ranges; SIS = skill item series; IQR = Interquartile range; BMI = Body Mass Index; SLJ = Standing Long Jump; * significant difference between the motor competence groups (>P15 and ≤P15) shown by the Mann-Whitney-U test. Underlined values indicate a significantly different distribution of the children across the motor competence groups per country. Underlined values indicate a significantly different distribution of the children across the motor competence groups per country

**Table 4:** Odds ratios for predicting low motor competence (≤percentile 15).

	Predictor	Odds Ratio’s		Significance	Interpretation
		Mean	95% CI		Probability for Having Low Motor Competence
**Static balance SIS**					
▪ Ghanaian group	BMI	1.228	[1.101; 1.371]	*p* < 0.001	A 1-point BMI ↑ → ↑ probability of 22.8%
	SLJ	0.970	[0.949; 0.992]	*p* < 0.001	A 10-cm SLJ ↓ → ↑ probability of 30.0%
▪ South African group	BMI	1.079	[1.026; 1.136]	*p* = 0.003	A 1-point BMI ↑ → ↑ probability of 7.9%
	SLJ	0.987	[0.978; 0.996]	*p* = 0.005	A 10-cm SLJ ↓ → ↑ probability of 13.0%
**Dynamic balance SIS**					
▪ Ghanaian group	BMI	1.106	[1.022; 1.198]	*p* = 0.013	A 1-point BMI ↑ → ↑ probability of 10.6%
	SLJ	0.975	[0.963; 0.988]	*p* < 0.001	A 10-cm SLJ ↓ → ↑ probability of 25.0%
▪ South African group	BMI	1.122	[1.063; 1.184]	*p* < 0.001	A 1-point BMI ↑ → ↑ probability of 12.2%
	SLJ	0.984	[0.975; 0.993]	*p* = 0.001	A 10-cm SLJ ↓ → ↑ probability of 16.0%
**Jumping and hopping SIS**					
▪ Ghanaian group	BMI	1.141	[1.046; 1.245]	*p* = 0.003	A 1-point BMI ↑ → ↑ probability of 14.1%
	SLJ	0.965	[0.950; 0.980]	*p* < 0.001	A 10-cm SLJ ↓ → ↑ probability of 35.0%
	Urban area	3.364	[1.417; 7.988]	*p* = 0.006	Three times more likely
▪ South African group	BMI	1.160	[1.098; 1.230]	*p* < 0.001	A 1-point BMI ↑ → ↑ probability of 16.0%
	SLJ	0.985	[0.975; 0.994]	*p* = 0.002	A 10-cm SLJ ↓ → ↑ probability of 15.0%
	Urban area	2.785	[1.602; 4.844]	*p* < 0.001	Three times more likely

Legend: BMI = Body Mass Index (kg/m^2^); SLJ = Standing Long Jump (cm). ↑ = increase, ↓ = decrease

## Data Availability

The data presented in this study are available on request from the corresponding author. The data are not publicly available due to privacy.

## Supplementary Materials

The following are available online at https://www.mdpi.com/article/10.3390/ijerph18157878/s1, Table S1: Differences in motor competences between boys and girls across different age groups. Figure S1: Median jumping and hopping SIS for boys and girls across age groups
