# Supplementary material for: High BMI and Low Muscular Fitness Predict Low Motor Competence in School-Aged Children Living in Low-Resourced Areas
Source: Int J Environ Res Public Health. 2021 Jul 25;18(15):7878. doi: 10.3390/ijerph18157878 (PMC8345384; doi:10.3390/ijerph18157878)
Supplement: Supplementary file 1 [file ijerph-18-07878-s001.zip › ijerph-1278350-supplementary.pdf]

# Supplementary materials

**Table S1**: Differences in motor competences between boys and girls across different age groups

| Age groups | Motor competence | Independent Mann-Whitney U test: p-value |
|---|---|---|
| 6 | Static Balance | .254 |
| | Dynamic Balance | .816 |
| | Jumping & hopping | .530 |
| 7 | Static Balance | .606 |
| | Dynamic Balance | .636 |
| | Jumping & hopping | .949 |
| 8 | Static Balance | .209 |
| | Dynamic Balance | .496 |
| | Jumping & hopping | .040 |
| 9 | Static Balance | .368 |
| | Dynamic Balance | .435 |
| | Jumping & hopping | .942 |
| 10 | Static Balance | .513 |
| | Dynamic Balance | .366 |
| | Jumping & hopping | .540 |
| 11&12 | Static Balance | .987 |
| | Dynamic Balance | .626 |
| | Jumping & hopping | <.001 |

**Figure S1**: Median jumping and hopping SIS for boys and girls across age groups

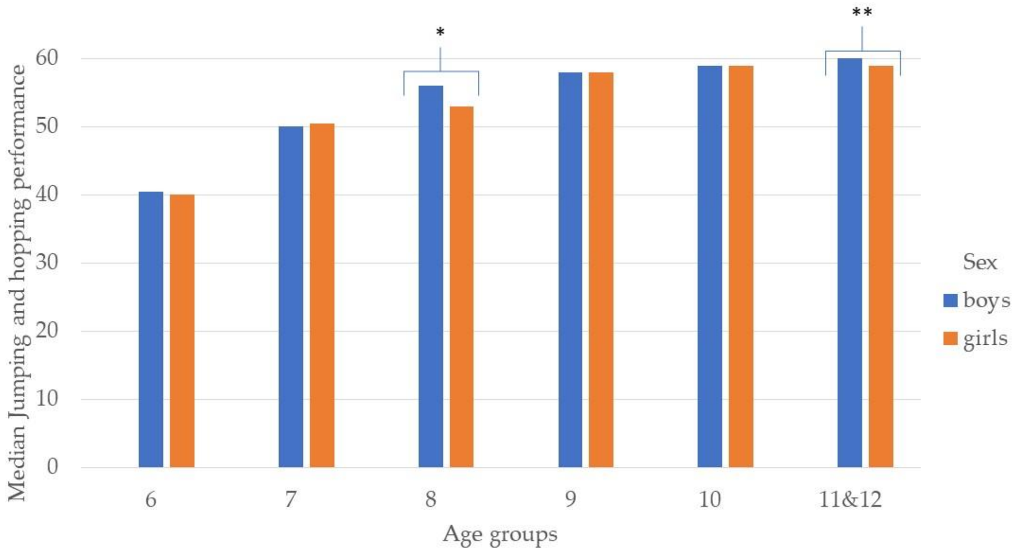


* Significant difference between boys and girls ($p<0.05$)
** Significant difference between boys and girls ($p<0.001$)
